# Supplementary material for: Inferring Social Status and Rich Club Effects in Enterprise Communication Networks
Source: PLoS One. 2015 Mar 30;10(3):e0119446. doi: 10.1371/journal.pone.0119446 (PMC4379184; doi:10.1371/journal.pone.0119446)
Supplement: S1 Text — (PDF) [file pone.0119446.s001.pdf]

S1 Text. Inferring social status and rich club effects in enterprise communication networks.

Yuxiao Dong[1], Jie Tang[2], Nitesh V. Chawla[1,*] Tiancheng Lou[3], Yang Yang[1], Bai Wang[4]
1 Interdisciplinary Center for Network Science and Applications, Department of Computer Science and Engineering, University of Notre Dame, Notre Dame, IN, United States of America
2 Department of Computer Science and Technology, Tsinghua University, Beijing, P. R. China
3 Google Inc, Mountain View, CA, United States of America
4 Department of Computer Science and Technology, Beijing University of Posts and Telecommunications, Beijing, P. R. China
∗ E-mail: nchawla@nd.edu

Prediction Experiment Setup

We consider the three different networks – CALL, SMS, and EMAIL – to evaluate the effectiveness of the model in predicting social status. We derive a number of different features from the data to build the training set of the machine learning algorithm. These include:
Feature Definition. There are mainly two types of features. The first type is mobile communication feature (domain specific). For example, a feature for one individual can be the number of people she calls. Table 1 lists a detailed definition of 10 communication features used in the baseline models and the proposed models. The second type includes social features that are defined based on social theories considered in the paper.

- **Structural hole:** The structural hole feature is determined by the score that one individual is spanned as structural hole.
- **Link homophily:** We simply use the count of the common neighbors between two individuals in the network to represent homophile. Note we are using the number of common neighbors as a model level attribute to infer homophile.
- **Social balance:** We derive four real-valued features to represent the proportions of the four types of (un)balanced triangles in the network.
- **Social clique:** We define a group (binary) feature for those users who belong to the same social clique or not (1 or 0).

Table 1. Communication features defined for node v in the communication networks.

Feature	Description
$k_{cin}(v)$, $k_{cout}(v)$	CALL in- and CALL out- degree
$t_{cin}(v)$, $t_{cout}(v)$	CALL in- and CALL out- event
$d_{cin}(v)$, $d_{cout}(v)$	CALL in- and CALL out- duration
$k_{sin}(v)$, $k_{sout}(v)$	SMS in- and SMS out- degree
$t_{sin}(v)$, $t_{sout}(v)$	SMS in- and SMS out- event
$k_{ein}(v)$, $k_{eout}(v)$	EMAIL in- and EMAIL out- degree
$t_{ein}(v)$, $t_{eout}(v)$	EMAIL in- and EMAIL out- event

Comparison Methods. We compare our proposed models with the four baseline methods: Naive Bayes (NB), Bayes Network (BNET), Logistic Regression Classification model (LRC) and Conditional

Random Fields (CRF) [1]. We use Weka[1] for NB, BNET and LRC methods. NB, BNET and LRC use communication attributes to train classification models and apply them to predict individual status and signs of social ties. For CRF and our proposed two models, both communication and social features are used to infer the labels of nodes and edges.

Evaluation Metrics. We quantitatively evaluate the performance of inferring the type of social relationships and individual status in terms of weighted *Precision, Recall, F1-Measure* and *Accuracy.*

Our models are implemented in C++, and all experiments are performed on a server running Apple OS X Sever with quad-core Intel(R) CORE i7 CPU @2.60GHz (4 CPUs) and 16GB memory. Basically, we simulate the prediction process 100 times for each algorithm on each dataset, randomly re-choosing 10% of the data as training set and the rest 90% as test set. The standard deviations of all prediction results are less than 0.05. The learning algorithm can converge in less than 20 iterations in all cases.

How Do Social Theories Help Reveal Social Status?

We now analyze how different social theory factors can help infer individual status. We consider five social factor functions: structural hole (SH), social tie (ST), social clique (SC), link homophily (HO) and social balance (SB). First, we remove each particular factor from our model and evaluate the decrease of the prediction performance in terms of *F1-Measure* by FGM model. A larger decrease means a higher predictive power.

Figure 1 A shows the average *F1-Measure* over CALL network, obtained by FGM. In particular, NSFG represents that we use all the social factors(structural hole, social tie, social clique, link homophily and social balance). It is obvious that the performance drops when ignoring each of these factors. We can see that for inferring status, the social balance theory factor is more important than other factors, because NSFG-SB drops more sharply than others. Link homophily factor contributes in the second place and structural hole factor takes the least contributions to infer social status. Then we consider the factor contributions by adding factors to the basic NSFG model (BASE) which only uses the communication features to train the model. In Figure 1 B, we find NSFG+SB increases most significantly in terms of *F1-measure*, compared with others, which means social balance factor is the most influential factor for both cases. Basically, the quantity of contribution each factor makes in BASE+X coincides with the NSFG-X, including SH, SC, ST, SB. However, BASE+HO model does not show comparable contributions in removing case (NSFG-HO) compared with SC factor, because there should exist correlations between different social factors. The factor contribution analysis validates that our method works well when combining different social theories and each social factor in our models contributes to the improvement of performance.

Call Duration vs. Social Status

We also examine the call duration [2] and try to understand two questions. One is how the call duration reflects different properties of social ties between staff with different status, the other is how the duration distributions depend on the status of the communication users. From Figure 2 A, we find that the average duration, which happens between two managers is the highest among four cases. If a subordinate calls a manager, it lasts a shorter duration. Figure 2 B and C tell us the similar pattern with tie duration, which is that the calls made by managers are a little bit longer than subordinates. Basically, the difference on call duration between managers and subordinates is not that obvious when comparing with communication attributes mentioned above.

[1]http://www.cs.waikato.ac.nz/ml/weka/

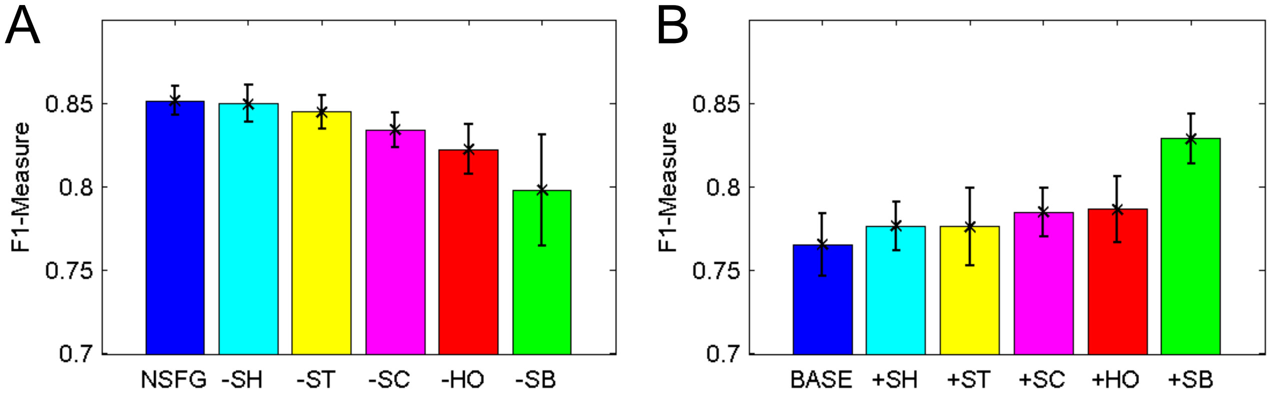

Figure 1. Factor Contribution Analysis in CALL network. (A). FGM means our proposed original factor graph model. -SH stands for ignoring structural hole correlation. -ST stands for ignoring social tie correlation. -SC stands for ignoring the correlation of social clique. -HO stands for ignoring link homophily. -SB stands for ignoring social balance. (B) BASE stands for all social factors mentioned above, and only takes communication attributes into consideration. '+' stands for adding a social factor to the BASE model.

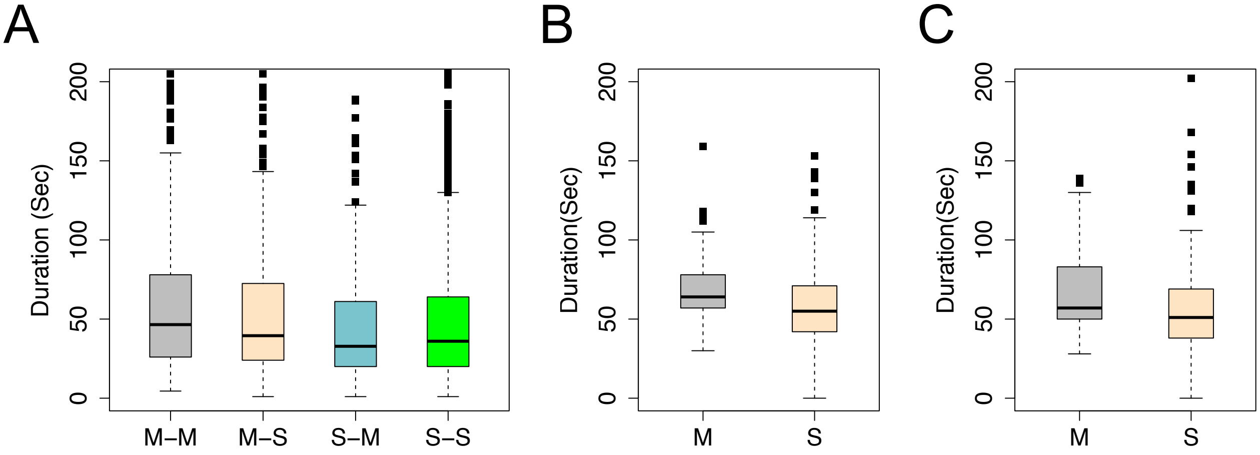

Figure 2. CALL Duration vs. Social Status. (A). Call duration between two staff of different status; (B). Call in-duration of different status; (C). Call out-duration of different status.

References

1. Lafferty JD, McCallum A, Pereira FCN (2001) Conditional random fields: Probabilistic models for segmenting and labeling sequence data. In: ICML'01. pp. 282–289.

2. Dong Y, Tang J, Lou T, Wu B, Chawla N (2013) How long will she call me? distribution, social

theories and duration prediction. In: ECML/PKDD'13. pp. 16-31.
